# Successful Treatment of* Klebsiella pneumoniae* Harboring a* Klebsiella pneumoniae* Carbapenemase Isolated from Lumbar Wound Infection and Blood in a Patient with Hardware Retention

**DOI:** 10.1155/2017/9028543

**Published:** 2017-09-17

**Authors:** Alan Bulbin, Carol Bono, Tena Philp, Noriel Mariano, Carl Urban

**Affiliations:** ^1^Division of Infectious Diseases and Antimicrobial Stewardship, St. Francis Hospital, Roslyn, NY, USA; ^2^The Dr. James J. Rahal, Jr. Division of Infectious Diseases, Department of Medicine, NewYork-Presbyterian/Queens, Flushing, NY, USA

## Abstract

Infections caused by carbapenem-resistant Enterobacteriaceae, especially carbapenemase producing* Klebsiella pneumoniae*, represent an urgent threat as outlined by the Centers for Disease Control and Prevention (CDC). We present a 66-year-old male with spinal stenosis who underwent elective L2-pelvis posterior spinal fusion at an outside institution and rapidly developed a complicated infection with* Klebsiella pneumoniae* harboring* Klebsiella pneumoniae* carbapenemase. This is the first described case of a patient with* Klebsiella pneumoniae* harboring* Klebsiella pneumoniae* carbapenemase causing postoperative lumbar wound infection and bacteremia, successfully treated with ceftazidime-avibactam in combination with additional synergistic antibacterials and without hardware removal.

## 1. Introduction

In 2013, the CDC listed carbapenem-resistant Enterobacteriaceae (CRE) as an urgent threat [1]. Infections caused by CRE, especially those producing carbapenem-hydrolyzing enzymes, are often extremely difficult to treat. In the United States, the majority of infections caused by CRE harbor* Klebsiella pneumoniae* carbapenemases (KPCs) and almost half of the hospitalized patients with carbapenem-resistant* Klebsiella pneumoniae* with KPCs (CRKP) bacteremia die from their infection [2]. The optimal treatment regimen for CRKP bacteremia and other complicated infections has not been established [2–4]. Combination therapies with carbapenems, polymyxins, aminoglycosides, tigecycline, and rifampin have all been reported [2–5]. We present the first reported case of an infected patient with* Klebsiella pneumoniae* harboring KPC enzymes, who developed postoperative lumbar wound infection and bacteremia, treated with ceftazidime-avibactam in combination with polymyxin B and rifampin and without removal of associated hardware.

## 2. Case Presentation

This is a 66-year-old, 81 kg male with spinal stenosis, chronic back pain, remote L4-L5 laminectomy, diabetes, and hypertension, who underwent elective L2-pelvis posterior spinal fusion on June 25, 2015, at an outside institution. The immediate postoperative course was notable for hypotension, asystole, cardiac arrest, acute myocardial infarction, and respiratory failure. He was resuscitated promptly but required further recovery in the intensive care unit. As of July 4, he was transferred to our institution for tertiary cardiac evaluation.

**Figure 1:**
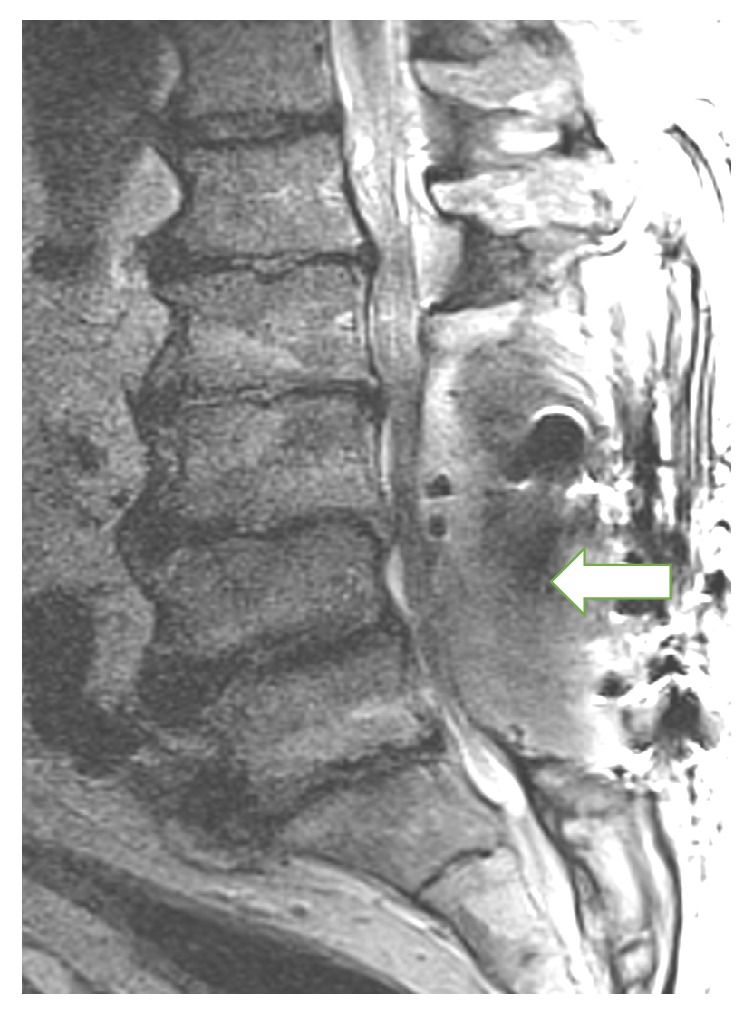
Lumbar spine MRI from July 6; arrow designates 12 × 3 × 3 cm abscess posteriorly with severe cord compression of the thecal sac, most prominent at the L4-L5 level (T2-weighted image provided by Craig Sherman).

After arrival, on July 6, the patient developed fever, prompting blood cultures and magnetic resonance imaging (MRI) of the lumbar spine. Piperacillin-tazobactam (Pip-Tazo) 3.375 g IV was administered by extended infusion over 4 hours TID as empiric coverage. Images (Figure 1) revealed posterior spinal fusion with rods and screws L2-S1, along with a 12 × 3 × 3 cm fluid collection posteriorly with severe cord compression of the thecal sac, most prominent at the L4-L5 level. Blood cultures, in single aerobic bottle (1 of 4 bottles), grew* Klebsiella pneumoniae*.

Antibiotic sensitivities were performed in the Clinical Microbiology Laboratory using a Microscan® system with Gram-negative Combo 34 panels (Beckman Coulter, USA). Minimal inhibitory concentrations (MICs) for ertapenem, imipenem, and meropenem were >4 *µ*g/ml, >8 *µ*g/ml, and >8 *µ*g/ml, respectively. Additional MICs were 3 *µ*g/ml for polymyxin B and 3 *µ*g/ml for tigecycline by E-test methodology according to the manufacturer's specifications (bioMérieux, Durham, NC).

The stored frozen isolate was later tested in the Infectious Diseases Research Laboratory for detection of beta-lactamases using the Check-MDR CT103 XL microarray system according to the manufacturer's instructions (Check-Points, Wageningen, Netherlands). Beta-lactamases identified using this system were KPC beta-lactamase (confirming CRKP), with additional enzymes including TEM (WT beta-lactamase) and SHV (238S and 240K mutations, extended-spectrum beta-lactamase).

Based on the above MRI findings, the patient was taken to the operating room (OR) on July 7. He underwent evacuation of the epidural collection, lysis of adhesions with foraminotomies, and wound revision. Hardware was left in situ. All operative cultures grew CRKP.

Pip-Tazo was continued postoperatively until the above sensitivities were known. As of July 11, treatment was switched to the triple combination of meropenem (1 g IV TID), tigecycline (50 mg IV BID, after a 100 mg loading dose), and polymyxin B (500,000 units IV BID). His 81 kg weight was used to establish CrCl and weight based dosing of polymyxin B. However, with rapid rise in creatinine, polymyxin B was discontinued on July 13. The double combination of meropenem and tigecycline was subsequently maintained without obvious adverse effect for the next 23 days. He initially did well and was able to complete his cardiac workup. All subsequent blood cultures remained negative throughout the remainder of his hospital stay. However, lumbar wound dehiscence later emerged.

On August 3, drainage from the patient's lumbar wound was first described. As of August 6, gross wound dehiscence prompted return to the OR, deep wound exploration, and evacuation of another encountered fluid collection. The fascia was noted to be dehisced, along with a fluid collection extending into the inferior portion of the surgical bed with exposed hardware. The inferior hardware was described to be completely surrounded by fluid. No cerebral spinal fluid leak was found. The area was irrigated copiously and complex closure was performed. All hardware was once again left in situ.

All wound cultures obtained on August 3 and at the time of surgery on August 6 again grew CRKP. MICs to the carbapenems were the same as the initial blood isolate and were 0.5/4 *μ*g/ml to ceftazidime-avibactam (Caz-Avi) (performed via Allergan® Reference Lab, 1651-A Crossings Pkwy., West Lake, Ohio 44145).

As of August 7, the patient's antibiotic regimen was switched to the triple combination of polymyxin B, adjusted for creatinine clearance (CrCl), 500,000 units IV BID, rifampin 300 mg PO BID, and Caz-Avi (also renal-adjusted) 1.25 g IV TID. This triple combination was maintained for the next 25 days; however, with progressive reduction in CrCl, polymyxin B was discontinued, followed later by rifampin. Caz-Avi was continued as single therapy for an additional 10 days, completing 6 weeks total of antibiotics from the time of the patient's second surgery. The decision was made to discontinue all antibiotics as of September 17. During this 6-week interval, the patient's back pain slowly improved. He was able to participate in physical therapy with a steady increase in exercise tolerance.

**Figure 2:**
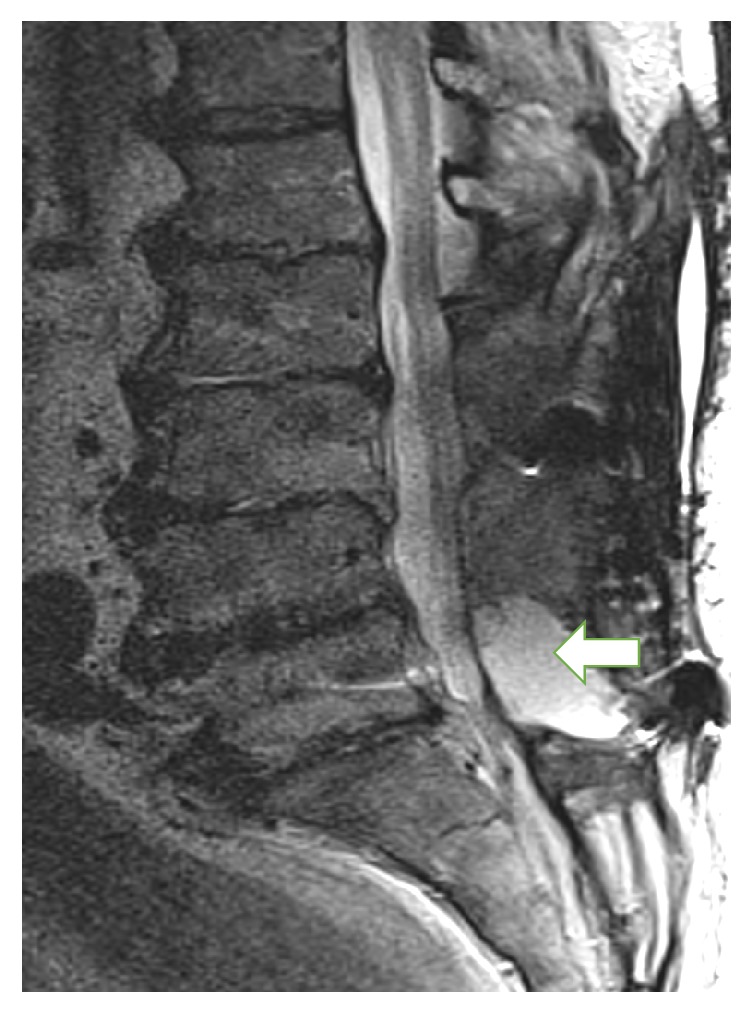
MRI from October 9; arrow designates a 3.5 cm fluid collection posterior to the thecal sac at the L5 level; aspiration proved sterile (T2-weighted image provided by Craig Sherman).

**Figure 3:**
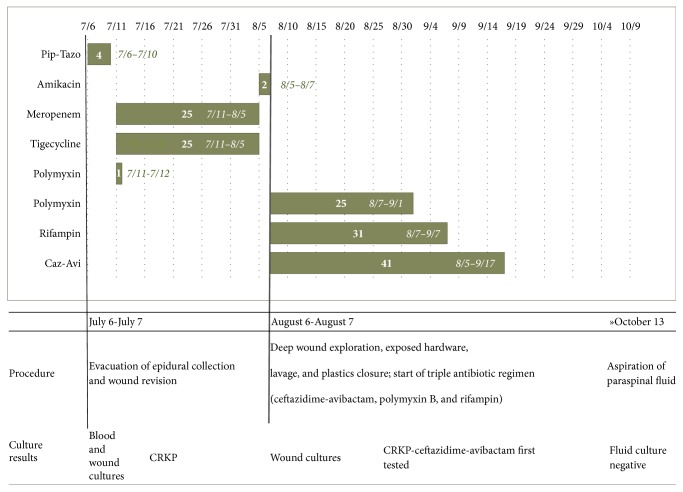
Culture results, procedures, and timing of antibiotic administration.

In view of persistent orthostatic hypotension, a follow-up MRI was performed. On October 9, images revealed no evidence of discitis or osteomyelitis; however, posterior to the thecal sac at the L5 level, there was a 3.5 cm fluid collection (Figure 2). As of October 13, this collection was aspirated via CT guidance; four milliliters of clear, yellow fluid was obtained by the interventional radiologist. This specimen proved to be sterile, with no growth after 5 days of incubation.  Figure 3 summarizes the patient's hospital course with timing of antibiotic, procedure, and culture results at our institution. No further imaging or cultures were performed after October 13, and in view of his overall clinical stability, the patient was discharged to a rehabilitation facility on October 27. Follow-up with the patient subsequently at 6, 12, and 18 months after discharge revealed steady clinical improvement. The patient admitted to feeling well and was pain-free and ambulating without assistance. He denied any further problems with the lumbar incision and required no further surgical intervention.

## 3. Discussion

To the authors' knowledge and after a literature review (PubMed, Medline, and Google Scholar), we believe that this case represents the first report of ceftazidime-avibactam use in conjunction with additional antibiotics for CRKP bacteremia and wound infection following lumbar fusion surgery. This challenging case highlights several important features. First, there is historical significance, as our patient received ceftazidime-avibactam only 3 months after the drug's release. The antibiotic regimen initiated prior to ceftazidime-avibactam's addition was indicative of the more typical approach in treating patients infected with bacteria harboring KPCs [2–4]. However, using older drug combinations has shortcomings, including baseline resistance and increased toxicity, which were both encountered in our patient's and other cases. Finally, a surprising feature of this case relates to the fact that the lumbar wound infection was successfully eradicated without hardware removal.

The initial treatment plan was to relegate the patient to an extended course of triple therapy with the combination of meropenem, tigecycline, and polymyxin B, as previously described [6, 7]. This was promptly abandoned for the combination of meropenem and tigecycline in view of rising creatinine, attributable to polymyxin B. Meropenem and tigecycline likely were inferior due to rapid hydrolysis of meropenem by the KPC, possible upregulation of efflux pumps, and downregulation of porin proteins [8–12]. However, variability in the interpretation of the corresponding MICs by E-test methodology for wound and blood isolates, as well as diffusion problems with cationic peptides, cannot be ruled out.

Ultimately, the patient failed initial surgical evacuation of the epidural abscess on July 7 followed by treatment with longer than 3 weeks of meropenem and tigecycline.

Subsequently, with sampling of wound drainage from August 3, his CRKP isolate was first tested and found to be susceptible to ceftazidime-avibactam. The decision to combine this, along with polymyxin B and rifampin, while accepting the relative problems with polymyxin B as previously observed, was based on the benefit of rifampin's use in the setting of infected hardware, lack of efficacy when used alone, and synergy with cationic peptides. Rifampin also provides the advantage of excellent penetration into abscess and biofilms. This strategy has been reported most often in staphylococcal infections [13, 14]. The benefit of rifampin when added with polymyxin B or colistin for synergy against CRKP and other carbapenem-resistant Gram-negative isolates has also been described [15, 16]. The use of both rifampin and polymyxin B in this case, along with ceftazidime-avibactam in combination, may have provided the ideal spectrum for successful hardware retention and lack of progression to ceftazidime-avibactam resistance, which has been observed with this agent alone, or even before exposure to the drug, when treating KPC producing organisms [8, 17]. Whether ceftazidime-avibactam used alone would have led to this same outcome is difficult to conclude but was not considered, especially since publications have cautioned against its use as a single agent, which has led to resistance while on therapy [8, 9]. Sampling of the small amount of paraspinal fluid seen on subsequent MRI as of October 9, roughly 6 weeks after completion of all antibiotics, proved it to be sterile. Although sedimentation rate and C-reactive protein were not followed up postoperatively, the facts that the posttreatment aspiration was sterile, the collection significantly reduced in size on follow-up MRI, and lumbar symptoms resolved were considered the best correlates for clearance of infection during his hospital stay. The patient showed no signs of recurrent infection greater than one year after completion of therapy.

Even with the availability of a singularly active agent like ceftazidime-avibactam, the ideal treatment regimen against KPC producing bacteria causing complicated wound infections and bacteremia still requires further investigation [11]. There is a growing literature showing that combination therapy may be better for multidrug resistant bacteria, especially when KPCs are implicated [3, 4, 10]. In the case presented here, a successful outcome was achieved utilizing ceftazidime-avibactam, polymyxin B, and rifampin.
